# Loss of NSE-4 Perturbs Genome Stability and DNA Repair in *Caenorhabditis elegans*

**DOI:** 10.3390/ijms23137202

**Published:** 2022-06-29

**Authors:** Arome Solomon Odiba, Chiemekam Samuel Ezechukwu, Guiyan Liao, Siqiao Li, Zhongliang Chen, Xihui Liu, Wenxia Fang, Cheng Jin, Bin Wang

**Affiliations:** 1 State Key Laboratory of Non-Food Biomass and Enzyme Technology, Guangxi Academy of Sciences, Nanning 530007, China; aromesolomonodiba@gxas.cn (A.S.O.); chiemekam.ezechukwu.pg76663@unn.edu.ng (C.S.E.); lsqgxas@163.com (S.L.); wfang@gxas.cn (W.F.); jinc@im.ac.cn (C.J.); 2 State Key Laboratory of Mycology, Institute of Microbiology, Chinese Academy of Sciences, Beijing 100101, China; 3 Department of Genetics and Biotechnology, University of Nigeria, Nsukka 410001, Nigeria; 4 Department of Zoology and Environmental Biology, University of Nigeria, Nsukka 410001, Nigeria; 5 Guangxi Key Laboratory of Sugarcane Genetic Improvement, Sugarcane Research Institute, Guangxi Academy of Agricultural Sciences, Nanning 530007, China; czl_good2007@126.com (Z.C.); liuxihui@gxaas.net (X.L.)

**Keywords:** *C. elegans*, DNA repair, meiosis, mitosis, NSE-4, SMC-5/6 complex

## Abstract

The Structural Maintenance of Chromosomes (SMC) complex plays an important role in maintaining chromosome integrity, in which the SMC5/6 complex occupies a central position by facilitating mitotic and meiotic processes as well as DNA repair. NSE-4 Kleisin is critical for both the organization and function of the SMC5/6 complex, bridging NSE1 and NSE3 (MAGE related) with the head domains of the SMC5 and SMC6 proteins. Despite the conservation in protein sequence, no functional relevance of the NSE-4 homologous protein (NSE-4) in *Caenorhabditis elegans* has been reported. Here, we demonstrated the essential role of *C. elegans* NSE-4 in genome maintenance and DNA repair. Our results showed that NSE-4 is essential for the maintenance of chromosomal structure and repair of a range of chemically induced DNA damage. Furthermore, NSE-4 is involved in inter-sister repair during meiosis. NSE-4 localizes on the chromosome and is indispensable for the localization of NSE-1. Collectively, our data from this study provide further insight into the evolutionary conservation and diversification of NSE-4 function in the SMC-5/6 complex.

## 1. Introduction

In prokaryotes and eukaryotes, the genomic material must be properly organized within the cell according to the dynamic functional requirements to support fundamental activities such as those that allow for appropriate proliferation, growth, and adaptation to the environment [1,2,3]. The Structural Maintenance of Chromosome (SMC) complexes are indispensable for this process, being broadly implicated in various aspects of genomic functionality, ranging from genome organization and stability to cell cycle regulation (mitosis, meiosis) and DNA repair [4,5,6]. The SMC family consists of three complexes, including cohesin, condensin, and the SMC5/6 complex [7,8,9]. The cohesin complex is composed of the SMC1 and SMC3 proteins and is required for sister chromatid cohesion, as a defect in cohesin induces premature separation of sister chromatids in budding yeast [10]. The condensin is composed of SMC2 and SMC4, and is primarily responsible for chromosome compaction and condensation, with additional roles in gene silencing, X-chromosome dosage compensation, and chromatid individualization during cell division [11,12]. The SMC5/6 complex consists of SMC5, SMC6, and the non-SMC elements (NSE), among which NSE1, NSE2 (Mms21), NSE3 (MAGEG1), and NSE-4 are highly conserved across eukaryotes, whereas NSE5 and NSE6 are not known to be conserved at the level of DNA sequence, as they have been identified only in yeast, *Arabidopsis*, and humans [13].

The SMC5/6 complex is involved in DNA replication, maintenance of stalled replication fork (RF), and DNA repair by homologous recombination (HR) [14,15,16,17]. Particularly, this SMC5/6 complex plays critical roles in the stalled replication fork (RF), which include restraining RF regression, stabilizing stalled RFs, reinitiating collapsed RFs, and cushioning topological stress during RF progression [18]. In yeast and human studies, the SMC5/6 complex has been shown to utilize ATP to interconnect two DNA molecules in a topological entrapment, as defective SMC6 did not interact with the DNA strand, which led to cell death with observable accumulation of DNA damage [19]. Among the SMC5/6 complex, it has been gradually established that SMC5/SMC6 together with NSE-4 kleisin serves as both structural and functional core of this complex [7]. NSE-4 kleisin mediates the interactions between SMC5, SMC6, NSE1, and NSE3 [20], and the underlying mechanisms began to be revealed by recent studies. A study in fission yeast revealed that the N-terminal domain of the NSE-4 kleisin links directly to the neck region of SMC6, bridging it to the head region of SMC5, and the KITE protein (NSE1 and NSE3) can further stabilize the complex in the ATP-free conformation through binding to NSE-4 [21]. In *Arabidopsis thaliana*, there are two NSE-4 paralogs, NSE-4A and NSE-4B, likely resulted from the gene duplication and specification in evolution [22]. Both NSE-4A and NSE-4B are involved in the DNA damage response and repair, although they differ in their regulatory roles [22]. NSE-4A appears to assume more functions than NSE-4B, regulating several activities such as chromatin organization, mitosis, and meiosis in plant development. NSE-4a and NSE-4b/EID3 account for human NSE-4 kleisins with both N-terminal and C-terminal kleisin domains that interact with MAGE protein NSE3 [23,24]. EID1, EID2, and EID2b, sharing homology of N-terminal NSE-4 kleisin domain, can also interact with MAGE proteins [23,24]. However, the overall mechanistic understanding of how the SMC5/6 complex is organized and regulated in various functional contexts is lagging, especially the specific role of NSE-4, which is yet to be fully understood. It can be greatly explored by studying in different model organisms such as yeast and *Caenorhabditis elegans* [25,26]. Here, we demonstrated the role of the *C. elegans* NSE-4 in protecting genome integrity and facilitating DNA repair. We also dissected the distribution of NSE-4 and the interactions essential for organizing the SMC5/6 complex.

## 2. Results

### 2.1. nse-4 Mutants Exhibited Reduced Fecundity and Chromosome Defect at Diakinesis

**Figure 1:**
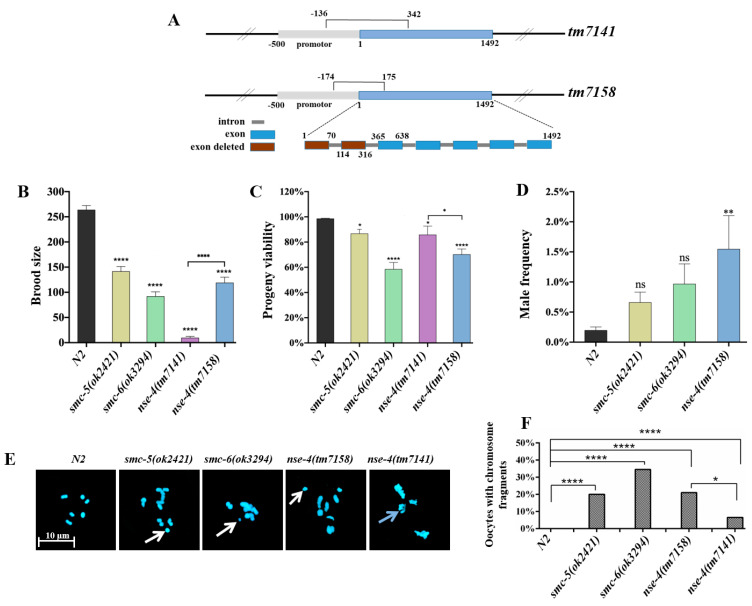
*nse-4* is important for fecundity and DSB repair. (**A**) Schematic representation of *C. elegans nse-4* gene structures in the mutants (*tm7141* and *tm7158*). In the *tm7141* allele, the gene segment between the promotor region (−136 bp) and exon 2 (342 bp) was deleted, while in the *tm7158* allele, the gene segment between the promotor region (−174 bp) and exon 2 (175 bp) was deleted. (**B**) Brood-size. (**C**) Progeny viability. (**D**) Male frequency. The symbol * on the top of the rectangle indicates differences with control N2, while the symbol * on the line shows the differences between 2 *nse-4* mutants. * *p* < 0.05, ** *p* < 0.01, **** *p* < 0.0001, and *p* > 0.05 (ns). (**E**) Representative micrographs of diakinesis chromosomes showing fragments (white arrows). *nse-4(tm7141)* is characterized by fragmented and fused chromosomes (blue arrow). Images were captured using Zeiss LSM800 Airyscan confocal microscope using 63× objective with oil immersion at a scale bar of 10 μm. (**F**) Percentages of diakinesis −1 and −2 oocytes containing chromosome fragments show that *smc-5/6* mutants have significantly higher frequency of chromosome fragmentation than the wild-type (**** *p* < 0.0001, * *p* < 0.05, chi-square Fisher’s Exact Test, *n* = 200 for each genotype). Oocytes were scored using Leica DM6 B at 100× objective with oil immersion.

NSE-4 is conserved in *C. elegans* (Figure S1), and two alleles of *C. elegans nse-4* mutant, *tm7141* and *tm7158,* were used in this study. In *tm7141*, nucleotides spanning the promoter (−136 bp) to CDS (342 bp) were deleted, whereas in *tm7158,* promoter (−174 bp) to CDS (175 bp) were deleted (Figure 1A). We observed 96% and 55% reduction in brood-size for *tm7141* and *tm7158*, respectively, as compared with the wild-type (**** *p* < 0.0001) (Figure 1B; Table S1). Furthermore, the viability showed a moderate reduction in both *tm7141* (13%, * *p* < 0.05) and *tm7158* (29%, ** *p* < 0.01), similar to the positive control *smc-5(ok2421)* and *smc-6(ok3294)* as compared with the wild-type (N2) (Figure 1C). Considering that *nse-4(tm7141)* had an average brood-size of about 10, it was surprising to observe 86% viability, which is higher than that of the *nse-4(tm7158)* allele with a higher brood-size of 119. In line with the brood-size analysis, *tm7158* allele exhibited a significantly higher male frequency (1.5%; ** *p* < 0.01) than the wild-type (Figure 1D), whereas no progeny from homozygous hermaphrodites of *nse-4(tm7141)* could develop to the adult stage for counting of males, suggesting severe disruptions in meiosis. Furthermore, no markable change in the number of GFP::COSA-1 foci were seen in *nse-4(tm7158)* (Figure S2), indicating that there was no defect in crossover (CO) recombination. 

To further investigate whether the above defect upon the loss of *nse-4* is related to compromised chromosome integrity, we carried out a cytological examination of the chromosome structure at diakinesis. There are six bivalent compact chromosomes with well-defined morphology observed in the -1 and -2 diakinesis oocytes in the wild-type [27]. As shown in Figure 1E,F, it was compromised in the SMC-5/6 complex mutants. Specifically, all the mutants displayed significant (**** *p* < 0.0001) chromosome fragments, whereas the *nse-4(tm7141)* additionally presented distinctive severe fusions of the chromosome (Figure 1E). The unexpected observations in the *nse-4(tm7141)* mutant at the phenotypic and chromosome levels prompted us to take a closer look at the internal architecture of the worm. We found that *nse-4(tm7141)* exhibited a smaller gonad, fewer germ cells and highly disorganized gonadal arrangements as compared with the wild-type, *nse-4(tm7158)*, *smc-5(ok2421)* and *smc-6(ok3294)* mutants (Figure S3).

### 2.2. Delayed Development in nse-4 Mutants Is Exacerbated by Genotoxic Stress

**Figure 2:**
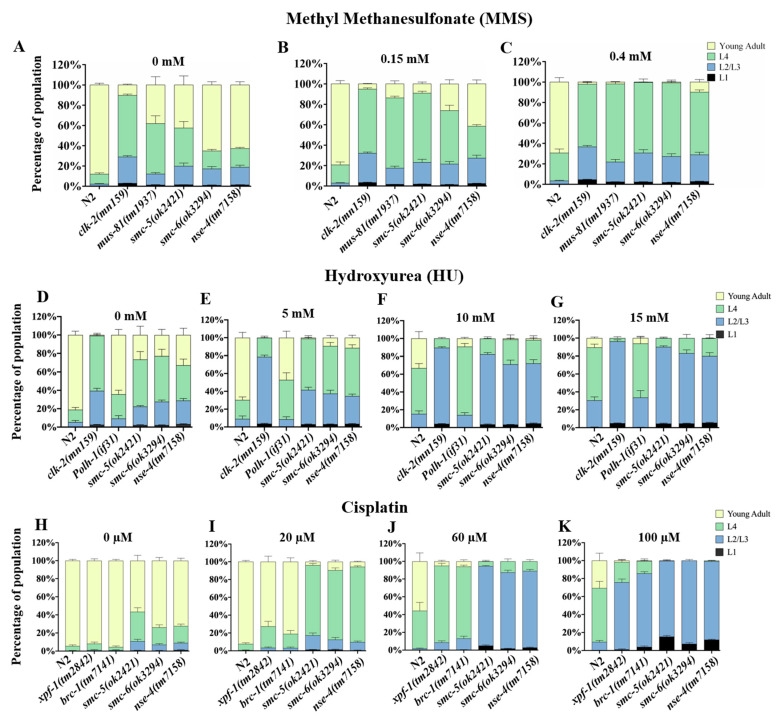
*nse-4(tm7158)* mutant exhibits developmental delay exacerbated by exposure to genotoxic stress. L1 stage worms were treated with different doses of DNA damaging agents for 16 h (20 h for HU) and allowed 48 h of recovery. (**A**–**C**) MMS. (**D**–**G**) HU, and (**H**–**K**) cisplatin. Results are cumulative of three independent repeats.

**Figure 3:**
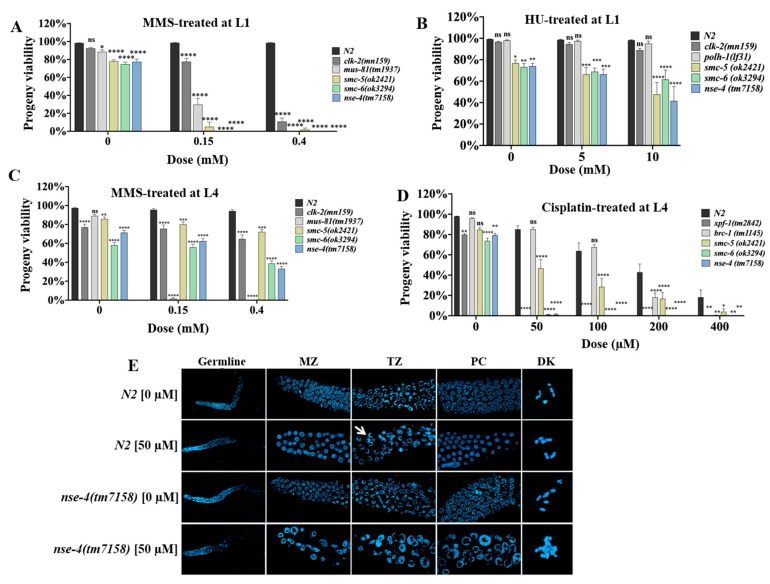
*nse-4 is* important for DNA repair. (**A**) Worm viability following exposure of L1 larvae to MMS at the indicated doses. (**B**) Worm viability following exposure of L1 larvae to HU at the indicated doses. (**C**) Worm viability following L4 stage worm exposure to MMS at the indicated doses. (**D**) Worm viability following L4 stage worm exposure to cisplatin at L4 stage. In all cases (**A**–**D**), viability was scored as the percentage of hatched eggs over the total number of eggs laid. Data represents cumulative of three independent repeats. The bar graphs show the cumulative mean ± S.E.M of worm viability compared to wild-type (ns—not significant; * *p* < 0.05; ** *p* < 0.01; *** *p* < 0.001; **** *p* < 0.0001, Two-way ANOVA with Dunnett’s multiple comparisons test). (**E**) Germline micrograph of cisplatin-treated worms. Worms were treated at a dose of 50 µM cisplatin for 16 h, allowed to recover on NGM plates for 24 h, and gonads were dissected and stained with DAPI. Germ cells are bloated and distorted, and the transition zone (white arrow) as well as chromosomal alignment at pachytene are absent in the cisplatin-treated *nse-4(tm7158)*. Whole germline (WG) was captured using Zeiss confocal microscope LSM 800 at 10× (scale bar = 50 μm); the other images were captured at 63× objective with oil immersion (scale bar = 10 μm). MZ—mitotic zone, TZ—transition zone, PC—pachytene, DK—diakinesis.

Animals deficient in DNA damage repair often exhibit delayed development and early death in response to genotoxic stress [28,29,30]. We exposed L1 stage animals to different doses of methyl methane sulfonate (MMS), hydroxyurea (HU), and cisplatin for 16 h and monitored their development by scoring the different larval stages after 48 h [31,32]. Different previously established positive controls strains were employed in the assays upon specific types of genotoxic reagents in this study (Figure 2 and Figure 3). The *mus-81(tm1937)* and *xpf-1(tm2842)* strains are defective in resolving meiotic recombination (HR) intermediates of DSBs. *clk-2(mn159)* is established in previous studies as sensitive to MMS as well as HU. The *brc-1(tm1145)* is deficient in inter-sister repair (HR). *lig-4(rb873)* is implicated in non-homologous end joining (NHEJ). *polh-1(if31)* is involved in the error-prone translesion synthesis. MMS is an alkylating agent, and DNA replication forks encountering the alkylated bases lead to DSBs [33]. The *nse-4(tm7158)*, *smc-5(ok2421)* and *smc-6(ok3294)* mutants were sensitive to MMS in a dose-dependent manner, which resulted in the developmental arrest at the L1/L2/L3 stages (Figure 2A–C; Table S2). Similar to the observation from MMS treatment, a dose-dependent developmental delay was seen in the worms treated with HU. HU inhibits DNA replication through the inhibition of ribonucleotide reductase, which is responsible for the synthesis of deoxyribonucleotides. The replication stress from HU causes the collapse of the fork to generate the DNA strand breaks during the S-phase [34,35]. We found that *nse-4(tm7158)* L1 worms were sensitive to HU with an enormous arrest at the early stages of development (Figure 2D–G; Table S3). In contrast to the wild-type strain, the *smc-5/6* mutants were mainly recorded as L1 stage worms at high doses. Cisplatin can introduce inter-strand cross links (ISCs) between two DNA strands, which are capable of blocking replication and causing fork stalling [36,37,38]. The *smc-5/6* mutants were sensitive to cisplatin in a dose-dependent trend (Figure 2H–K; Table S4). At the highest dose (100 µM), all the mutants tested were arrested at L1/L2 stages, whereas the wild-type were less affected (Figure 2K). The delay in the development was already obvious under physiological conditions (at 0 dose) in *nse-4(tm7158)*, *smc-5(ok2421)* and *smc-6(ok3294)* (Figure 2A,D,H, respectively), which suggests that *nse-4* mutants exhibit developmental defects even without genotoxic stress.

### 2.3. nse-4 Is Required for Efficient DNA Repair in Germ Cells

The DNA damage from genotoxic insults should be repaired by the cell. However, if left unrepaired, damaged cells either become apoptotic corpses or dead eggs after fertilization, resulting in reduced progeny survival [29,39,40,41]. To investigate the importance of *nse-4* in the repair competency, L1 worms were treated with MMS for 16 h, recovered for 72 h, and thereafter the viability of the eggs laid was scored [31,32]. The results showed that *nse-4(tm7158)* mutant was significantly (**** *p* < 0.0001) sensitive to MMS in a dose-dependent manner, as compared with the wild-type (Figure 3A; Table S5). Similarly, L1 worms treated with HU showed a significant decrease in the viability of the *nse-4(tm7158)* (**** *p* < 0.0001) and *smc-5(ok2421)* (*** *p* < 0.001) mutants at 10 mM dose as compared with the wild-type, while not significant for *smc-6(ok3294)* (*p* > 0.05) (Figure 3B; Table S6). The mutants appeared not to be significantly sensitive to HU at 5 mM, as the differences between 0 and 5 mM doses were not significant (*p* > 0.05). We did not carry out this assay for cisplatin as L1 worms exposed to a dose as low as 20 µM developed into sterile adults, while the worms exposed to higher doses failed to develop to the egg-laying stage (data not shown).

Likewise, we treated L4 worms with MMS and cisplatin for 16 h and scored the viability after 24 h of recovery [31,32,42]. Our results showed that, as compared with the wild-type strain, the *nse-4(tm7158)* allele already exhibited a significant (**** *p* < 0.0001) progeny viability defect even without treatment, similar to the *smc-6(ok3294)* mutants (Figure 3C; Table S7). However, *nse-4(tm7158)* showed a more significant defect (**** *p* < 0.0001) at 0.4 mM dose, when compared to the 0 mM dose of MMS (Table S7). Similarly, a highly significant (**** *p* < 0.0001) hypersensitivity to cisplatin even at a low dose (50 µM) was observed in the *smc-5/6* mutant worms (Figure 3D; Table S8). A relatively milder sensitivity was observed with the *smc-5(ok2421)*, as compared with *nse-4(tm7158)* and *smc-6(ok3294)* animals.

The hypersensitivity of *nse-4(tm7158)* mutant to cisplatin prompted us to examine the chromosome architecture in the germline. In all the germline zones examined, the number of nuclei was significantly reduced upon cisplatin stress, and aberrant morphology was obvious in the enlarged and distorted nuclei, which is often associated with the damage induced by ICL agents (Figure 3E). The observation in the mitotic zone (MZ) could be attributed to cell cycle arrest [43]. Moreover, key features of the transition zone (crescent-shaped nuclei) and pachytene stage (thread-like) were also absent in the *nse-4(tm7158)* mutant due to cisplatin treatment. The findings indicate that *nse-4* is important for DNA repair in germ cells.

### 2.4. nse-4 Deficiency Led to Increase RAD-51 Accumulation

**Figure 4:**
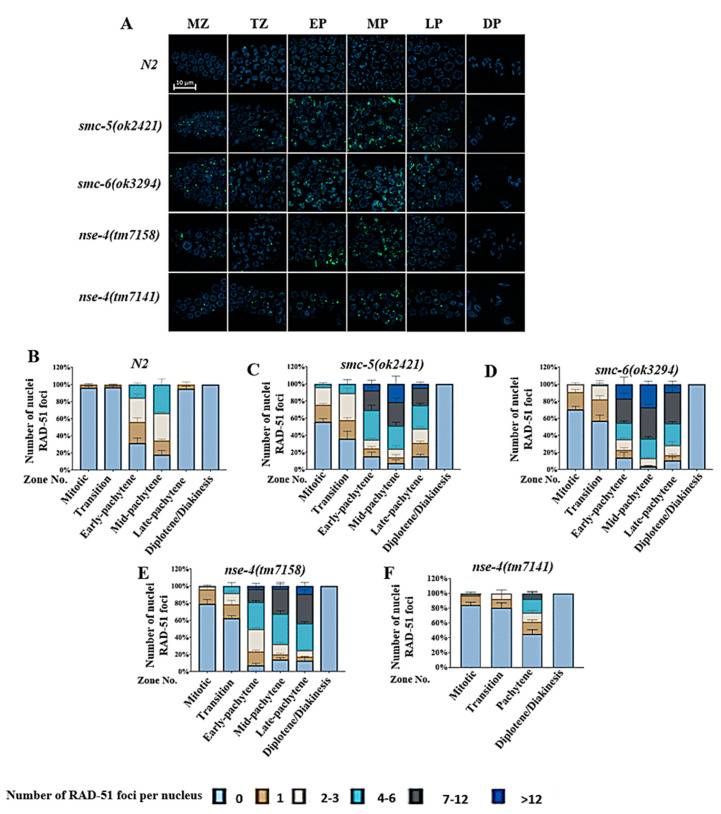
Abnormal RAD-51 accumulation in meiotic germ cells of *nse-4* mutants. (**A**) Micrographs of RAD-51 immunofluorescence and DNA-DAPI fluorescence at the different zones of wild-type and mutant germlines images were captured using a Zeiss confocal microscope LSM 800 with Airyscan at 63× objective with oil immersion (scale bar = 10 μm). (**B**–**F**) Distribution of RAD-51 foci in the germline is shown in the various zones of the gonad arms. Different colors represent the average number of RAD-51 foci per nucleus. Error bars represent standard error (±S.E.M).

We used RAD-51 immunostaining to quantify the unrepaired DSBs in the *nse-4* mutants [44]. In all zones from the mitotic zone (MZ) to late pachytene (LP), the numbers of RAD-51 foci were significantly higher in the *nse-4* mutants in contrast to the wild-type (Figure 4A–F; Table S9). We also noticed that the *nse-4(tm7141)* germline was smaller and distorted as compared with other genotypes. Although the MZ, transition zone (TZ), and diplotene zone (DP) can be carefully distinguished in *nse-4(tm7141)* worms, it is difficult to differentiate between early pachytene (EP), middle pachytene (MP) and late pachytene (LP). As a result, we could only quantify the pachytene area as a whole (Figure 4F). Whereas RAD-51 foci were rarely observed in the wild-type MZ and LP, their persistence in the *nse-4* mutants as consistently detected in *smc-5(ok2421)* and *smc-6(ok3294)* in previous studies suggests the role of the SMC-5/6 complex in the maintenance of chromosome integrity during homologous recombination (HR) [25]. The appearance and increased accumulation of RAD-51 foci in the unusual zones (MZ and TZ) of the *nse-4(tm7158)* and *nse-4(tm7141)* germlines suggest that the lack of *nse-4* could have stressed DNA processes such as replication and segregation [45]. Collectively, our results show that *nse-4* plays a significant role in the repair of DSBs.

### 2.5. nse-4 Mutants Are Defective in Inter-Sister Repair

**Figure 5:**
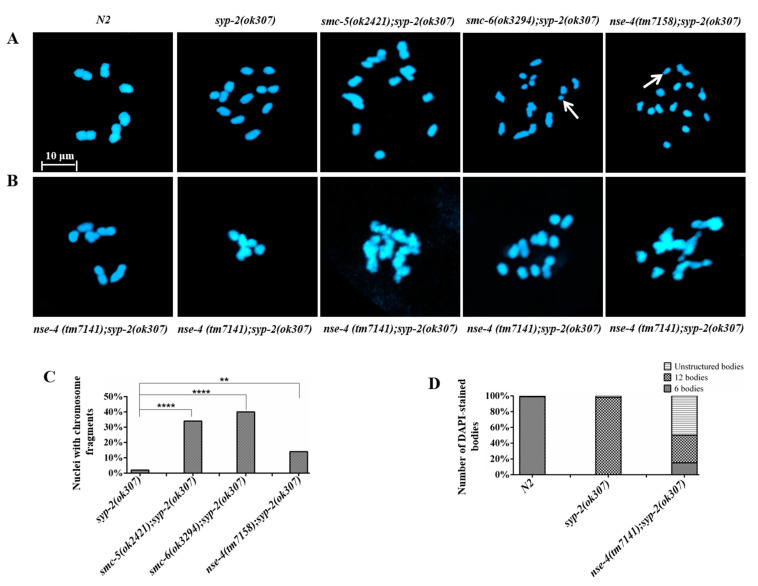
*nse-4* mutants showed defective inter-sister repair. (**A**) Micrographs of the diakinesis chromosome with fragments (white arrows) in the double mutants. (**B**) Representative micrographs showing chromosome structure at diakinesis in *nse-4(tm7158);syp-2(ok307)*. Chromosome bodies are grossly damaged, but surprisingly, some nuclei showed wild-type phenotype. (**C**) Quantification of chromosome fragments at diakinesis (** *p* < 0.01, **** *p* < 0.0001, chi-square Fisher’s Exact Test). The sample size (*n*) = 200 for each genotype. Chromosome fragments were scored using Leica DM6 B at 100× objective with oil immersion. (**D**) Quantification of DAPI-stained bodies of *nse-4(tm7141);syp-2(ok307)* diakinesis nuclei. *Nse-4(tm7141)* mutation rescued about 15% of the syp-2 diakinesis phenotype. The sample size (*n*) = 200 for each genotype. DAPI bodies were scored using Leica DM6 B at 100× objective with oil immersion. Images were captured using Zeiss confocal microscope LSM 800 with Airyscan at 63× objective with oil immersion (scale bar = 10 μm).

In *C. elegans*, several SPO-11 dependent DSBs per homologous chromosome are formed, but only one crossover (CO) event occurs during meiosis [46]. The remaining DSBs are repaired as non-CO by the inter-homolog or inter-sister recombination pathway [47,48,49,50]. A defect in this pathway leads to chromosomal fragmentation, which can be observed in the diakinesis nuclei [51,52]. *Syp-2* encodes a synaptonemal complex central element protein, which is required for bridging the axes of the paired meiotic chromosomes [53]. Homologous recombination repair is disrupted in *syp-2* mutant, but competent in inter-sister repair, which results in 12 DAPI-stained bodies (12 univalent chromosomes). To determine whether the chromosomal fragments observed in *nse-4* mutants are due to defects in inter-sister repair, we generated a series of double mutants including *nse-4(tm7158);syp-2(ok307)*, *nse-4(tm7141);syp-2(ok307)*, *smc-5(ok2421);syp-2(ok307)* and *smc-6(ok3294);syp-2(ok307)*, and quantified the DAPI-stained bodies in diakinesis oocytes. As compared with the wild-type, higher numbers of DAPI bodies (>12) were present in the *nse-4(tm7158);syp-2(ok307)* as well as *smc-5(ok2421);syp-2(ok307)* and *smc-6(ok3294);syp-2(ok307)* diakinesis nuclei (Figure 5A,B) [54]. The chromosomal fragmentation occurred with a much higher frequency (14%) in the *nse-4(tm7158)* as opposed to 2% in *syp-2(ok307)* (** *p* < 0.01), while there were 30% and 40% (**** *p* < 0.0001) for *smc-5(ok2421);syp-2(ok307)* and *smc-6(ok3294);syp-2(ok307)*, respectively (Figure 5C). Surprisingly, the *nse-4(tm7141)*;*syp-2(ok307)* double mutant displayed additional phenotypes that were not observed in *nse-4(tm7158);syp-2(ok307)*. Of all the oocytes checked, 15% showed 6 DAPI staining bodies, 35% showed 12 DAPI staining bodies, and 50% showed unstructured bodies (Figure 5B,D). Our data suggests that *nse-4* plays an important role during inter-sister recombination repair. 

### 2.6. Apoptosis Increased in the nse-4 Deficient Germ Cells

**Figure 6:**
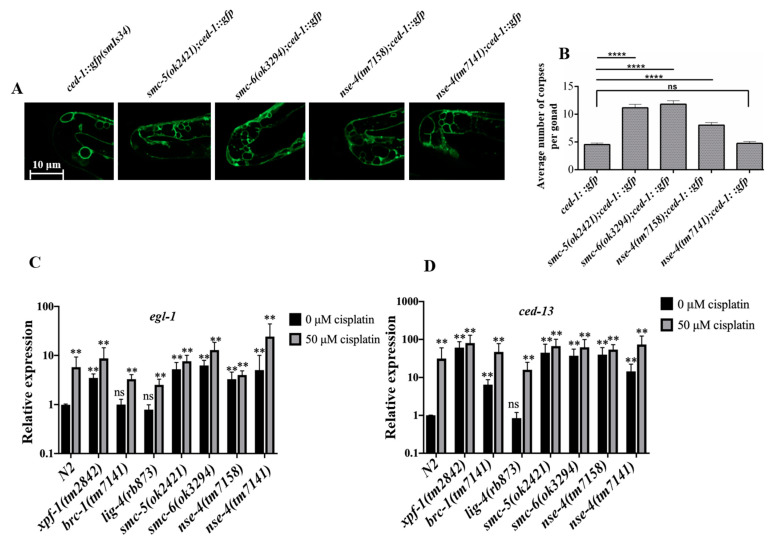
Mutations in *nse-4* lead to increased apoptosis in the *C. elegans* germline. (**A**) CED-1::GFP surrounds the apoptotic cells in the gonad arm and creates a ring of GFP fluorescence around dying cells. (**B**) Quantification of apoptotic corpses. The number of apoptotic cells significantly (**** *p* < 0.0001) increased in *smc-5(ok2421)*, *smc-6(ok3294),* and *nse-4(tm7158)* mutant background, but not significantly (*p* > 0.05) in *nse-4(tm7141)* background when compared to control (One-way ANOVA with Tukey’s multiple comparisons test, number of gonad arms scored for each strain, *n* = 50, ns = not significant). Images were captured using Zeiss confocal microscope LSM 800 with Airyscan at 63× objective with oil immersion (scale bar = 10 μm). Oocytes were scored using Leica DM6 B at 100× objective with oil immersion. (**C**) *egl-1* in the wild-type and mutant genotypes as measured by quantitative RT-PCR, and (**D**) relative mRNA abundance for *ced-13*. *Xpf-1(tm2842)*, *brc-1(tm1145)*, and *lig-4(rb873)* were used as control strains representing HR, inter-sister, and NHEJ pathways, respectively. Black and grey columns represent the relative expression levels (Means ± S. E. M) in different strains at 0 µM and 50 µM cisplatin doses, respectively, compared to wild-type at 0 µM cisplatin dose (unpaired two-tailed Student’s *t*-test, ** *p* ≤ 0.01 and ns—*p* > 0.05). The experiment was repeated twice, and all strains were independently analyzed in triplicate in each experiment.

Unrepaired DNA damage can induce cell death, which is detectable as apoptotic corpses [29]. Induction of apoptosis is mediated by the CEP-1/p53 pathway through the transactivation of two pro-apoptotic genes, *egl-1* and *ced-13* [55]. CED-1 is a transmembrane protein that functions in the engulfing (sheath) cells surrounding the surface of apoptotic cells. GFP tagged to CED-1 in the *ced-1::gfp(smIs34)* transgenic strain can serve as a ring marker for scoring cell corpses [54,56,57,58,59]. We crossed this strain to the various *smc-5/6* mutants, and the results showed that *nse-4(tm7158)*, *smc-5(ok2421),* and *smc-6(ok3294)* but not *nse-4(tm7141)* presented more apoptotic cells than the wild-type (Figure 6A). Quantification of this observation showed that the number of cell corpses was increased significantly (**** *p* < 0.0001) in *nse-4(tm7158)*, *smc-5(ok2421),* and *smc-6(ok3294)* mutants, but not significantly (*p* > 0.05) in *nse-4(tm7141)* (Figure 6B) compared to the wild-type. However, the organization of the *nse-4(tm7141)* germline is often compromised, which may have affected the result.

In *C. elegans*, both EGL-1 and CED-13 function in the cell death signaling pathway [58]. *egl-1* and *ced-13* function upstream of *ced-9*, *ced-4*, and *ced-3*, at a point where signals that specify the cell death fate are activated, and elevated levels indicate increased cell death [58]. We quantified the relative transcription of *egl-1* and *ced-13* using qRT-PCR analysis. Generally, *egl-1* mRNA levels were significantly (** *p* < 0.01) enhanced in the *smc-5/6* mutants as compared with the wild-type (Figure 6C; Table S10). The *ced-13* mRNA levels exhibited the same trend as *egl-1* (Figure 6D; Table S11). The elevated *egl-1* and *ced-13* mRNA levels in the *smc-5/6* mutants over the wild-type were consistent with the results of increased apoptotic cells (Figure 6B). Both *egl-1* and *ced-13* transcription in the *nse-4* mutants were further enhanced after 50 µM cisplatin treatment in comparison to the wild-type (Tables S10 and S11; Figure S4). The enhancement might be as a result of ICL-induced lesions that block DNA replication. Overall, the results demonstrated that the *nse-4* mutations resulted in increased apoptosis, which is dependent on the EGL-1 and CED-13 cell death signaling pathways.

### 2.7. NSE-4 Localizes on the Chromosome and Is Indispensable for the Localization of NSE-1

**Figure 7:**
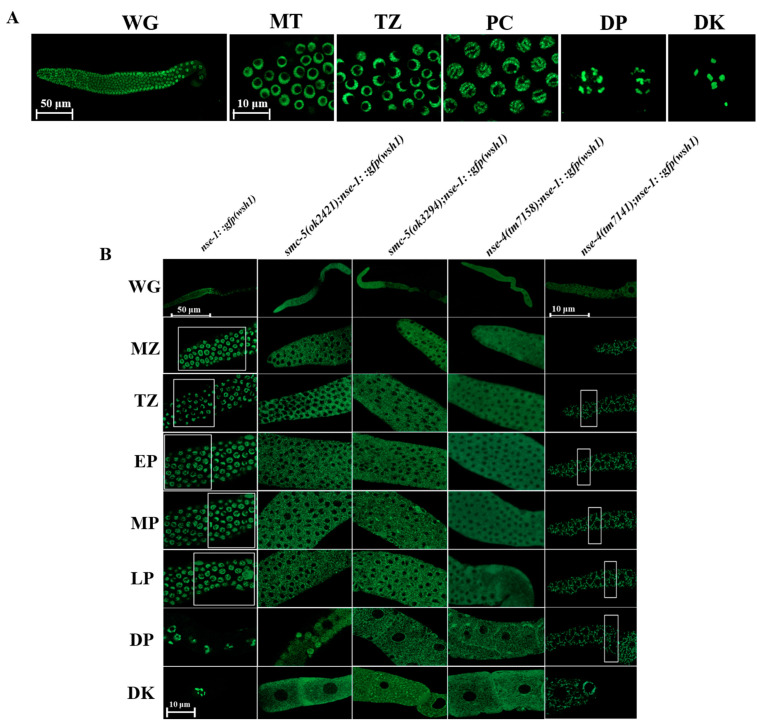
NSE-4 localizes on the chromosome and is necessary for NSE-1 localization. (**A**) NSE-4 localizes to the chromosome in all zones of the germline. (**B**) NSE-4 Kleisin is indispensable for the localization of NSE-1 on the chromosome. In *nse-4*, *smc-5*, or *smc-6* mutant background, NSE-1 delocalizes from the chromosome and is translocated to the cytoplasm in all zones of the germline. All images for whole germline (WG) were captured using Zeiss LSM800 confocal microscope with 10× objective and scale bar of 50 μm, except for the *tm7141;wsh1* with a remarkably small germline in which 40× objective with oil immersion was used at a scale bar of 10 μm. All other images of the different zones were captured using 63× objective with oil immersion and a scale bar of 10 μm (MZ—mitotic zone, TZ—transition zone, PC—pachytene, DP—diplotene, DK—diakinesis).

Studies in yeast, plants, and some animal models have contributed substantially to our current knowledge of the architecture of the Smc5/6 complex in eukaryotes [25,54,60,61,62]. Prior to this study, the localization of NSE-4 in the *C. elegans* model had not been characterized. We made a NSE-4::GFP transgenic worm by CRISPR-Cas9 system and found that NSE-4 localizes on the chromosome in all zones of the germline (Figure 7A). In fission yeast, NSE-4 serves as a central factor, interacting with NSE1, NSE3, the N-terminal domain of SMC5, and the C-terminal domain of SMC6 [63]. We crossed the transgenic strain *nse-1::gfp(wsh1)* to *smc-5/6* mutants, and examined the localization of NSE-1 in vivo. Our results showed that NSE-1::GFP localized on the chromosome at all stages in the wild-type germline, in a manner similar to the distribution pattern of NSE-4 (Figure 7B). However, the NSE-1 was delocalized from the chromosome and translocated into the cytoplasm at all zones of the germline in the *smc-5(ok2421)*, *smc-6(ok3294)*, *nse-4(tm7158),* and *nse-4(tm7141)* mutants. In the gonad of *nse-4(tm7141)*, which is relatively smaller than those of the other mutants, the translocated NSE-1 seemed to aggregate in the cytoplasm. Taken together, these observations suggest that NSE-4, SMC-5, and SMC-6 are indispensable for the localization of NSE-1 on the chromosome.

## 3. Discussion

Defects in genomic stability often lead to fertility deficiencies in animals [39,40,41]. As described in previous studies, the loss of the SMC-5/6 complex in *C. elegans* led to a reduction in brood-size as compared with the wild-type [25,54]. While most of the work done on NSE-4 was in yeast, plants and animal cells [20,21,22,61,64], there is no report on NSE-4 in *C. elegans* to date. In this study, we investigated the possible functions of *C. elegans* NSE-4 in ensuring genomic integrity. Two alleles of *C. elegans nse-4* studied here exhibited reduced fertility as reflected in the reduced brood-size and viability in comparison to the wild-type (Figure 1B,C). As compared to *tm7158*, the lower brood-size, higher progeny viability and lower frequency of chromosome fragments in oocyte for *tm7141* may be related to the larger deletion in the *tm7141* allele as depicted in Figure 1A. Generally, worms with a defect in meiosis usually exhibit mild to severe male frequency [27,65,66,67]. The significantly enhanced male frequency in the *nse-4(tm7158)* mutant may suggest a role in the meiotic chromosome segregation process. There are no previous studies implicating a significant increase in male frequency in any member of the *C. elegans* SMC-5/6 complex [25,54] (Figure 1D). As opposed to the fragments observed in the other mutants, the rather more fused and misshapen chromosomes in the *nse-4(tm7141)* allele diakinesis nuclei were probably due to defects in chromosome segregation (Figure 1E), which may provide evidence for an additional role for *nse-4* during meiosis. 

Defects in DNA damage repair lead to delayed growth, early death, or being arrested at the early developmental stages [28,29,30]. DNA replication is more active in the early development stages, particularly L1, and the unwinding of DNA is necessary for transcription by exposing DNA to polymerase activity [68,69]. Genotoxic agents often challenge this process, and if left unresolved, will hinder animal development [39,40]. The wild-type animals are competent at repairing the corresponding damage, thereby making them capable of restoring the development progress [31,70,71]. We performed DNA damage assays by exposing the L1 larva to MMS, HU, and cisplatin and observed a dose-dependent developmental arrest in the *nse-4* mutants as compared to the wild-type (Figure 2A–K; Table S2). This toxic effect is largely repaired through the DNA repair pathways [50,72,73,74]. Worms defective in this repair are sensitive to genotoxic drugs, which translate directly to developmentally arrested worms in the early stages [72]. However, the *nse-4* mutants demonstrated developmental defects without drug treatment (Figure 2A,D,H), suggesting that *nse-4(tm7158)* is crucial for the worm development. This developmental defect was further exacerbated upon exposure to genotoxic drugs, indicating that *nse-4* is essential for the survival of genotoxin-stressed cells. These results suggest that NSE-4 is vital for development as well as repair of DNA lesions in *C. elegans*.

Germ cells respond to DNA damage via the evolutionarily conserved classical DNA damage response (DDR) pathways in *C. elegans*. The cell cycle arrest of proliferating mitotic nuclei and the apoptosis of damaged nuclei make up spatially separate DDR checkpoints [39]. When the L1 larvae were exposed to genotoxic agents, the results showed that *nse-4(tm7158)* worms, similar to *smc-5(ok2421)* and *smc-6(ok3294)* mutants, are significantly (**** *p* < 0.0001) incompetent in repairing MMS-induced (Figure 3A; Table S5), and HU-induced stress (at 10 mM dose only) (Figure 3B; Table S6). In the L4 larvae assay, the *nse-4(tm7158)* mutant showed significantly (**** *p* < 0.0001) hypersensitive to all doses of cisplatin, and higher dose of MMS (Figure 3C,D; Tables S7 and S8, respectively). A previous study showed that the knockdown of human NSE-4a rendered the cells sensitive to MMS [24]. Similarly, studies in budding yeast *S. cerevisae* [75] and plant *A. thaliana* have reported the sensitivity of NSE-4 deficient cells to MMS, zebularine, and bleomycin (DSB-inducing agents) [22,61]. In the fission yeast *S. pombe*, NSE-4-defecient cells are hypersensitive to MMS and HU [21]. Bringing together all these results of the sensitivity of the *nse-4* mutants to genotoxic insults, it is apparent that *C. elegans nse-4* is pivotal for DNA damage repair.

Under normal physiological conditions in meiosis, DSB formation is executed by the SPO-11 topoisomerase-like proteins at meiosis entry [76,77]. After a 5′ to 3′ end resection of the single-strand, RAD-51 replaces the RPA-1 on the single-stranded DNA overhangs to promote the invasion of intact homologous DNA templates [78,79,80]. RAD-51 foci typically begin to appear at the transition zone (TZ), a point where meiotic DSB induction begins. As recombination advances, more RAD-51 foci are observed in the early-pachytene (EP) and peaks at the mid-pachytene (MP). All foci finally disappear at late-pachytene (LP) on completion of HR repair. More DSBs lead to increased RAD-51 foci and persistent unrepaired DSBs postpone the unloading of RAD-51 to later stages [81,82]. We observed an increase in the number of RAD-51 foci from MT to LP zones in the *nse-4* mutants over the wild-type animals (Figure 4A–F; Table S9). The appearance of RAD-51 foci in the mitotic area of *nse-4* mutants and its persistence into late pachytene strongly indicates a defect in the mitotic process and meiotic repair, respectively, pointing to its important role in ensuring the genome is stable. The RAD-51 abnormality detected in the *nse-4* mutants was similar to the observations made in *smc-5(ok2421)* and *smc-6(ok3294)* mutants, which are consistent with previous studies [25,45]. However, the *nse-4(tm7141)* germline was smaller and distorted as compared with *nse-4(tm7158)*. The difficulty to differentiate between EP, MP, and LP led to the RAD-51 quantifying as a whole in the pachytene area (Figure 4F), which could be caused by the larger deletion in *nse-4(tm7141)*. This similarity between these mutants could be attributed to the fact that proteins that form part of the same complex, or participate in the same pathway, are likely to have similar deleterious effects when any member is mutated [83].

In wild-type *C. elegans*, several SPO-11 dependent DSBs per homolog are generated but only one CO event per pair occurs [46], and the extra DSBs are repaired via inter-sister recombination repair as non-CO [47,48,49,50]. We proceeded to investigate whether *nse-4* is implicated in inter-sister repair during meiosis. We observed extra (14%) chromosomal fragments in *nse-4(tm7158);syp-2(ok307)* (Figure 5), as well as in *smc-5(ok2421);syp-2(ok307)* and *smc-6(ok3294);syp-2(ok307)*, consistent with previous studies [54]. As the synapsis was disrupted by *syp-2* mutation, chromosomal fragmentations could be observed at the diakinesis nuclei, suggesting that *nse-4* plays an important role during inter-sister repair [51,52,53]. The *nse-4(tm7141)*;*syp-2(ok307)* double mutant displayed additional phenotypes, such as 6 DAPI staining bodies and unstructured bodies (Figure 5B,D), which could result from the severe disruption of the germ cell. The number of cell corpses was increased significantly (**** *p* < 0.0001) in *nse-4(tm7158)*, *smc-5(ok2421)*, and *smc-6(ok3294)* mutants (Figure 6A,B), and *egl-1* and *ced-13* mRNA levels in *nse-4*, *smc-5,* and *smc-6* mutants were also significantly (** *p* < 0.01) enhanced (Figure 6C,D; Tables S10 and S11). The transcription level of *ced-13* and *egl-1* is related to apoptosis in germ cells [55,84,85,86]. The additional increase in the mRNA levels after cisplatin treatment could be a result of extra ICL-induced DNA damage. However, although the same *egl-1* and *ced-13* mRNA levels were detected, there are no significantly (*p* > 0.05) increased cell corpses in *nse-4(tm7141)* (Figure 6B). The cell corpses could be too severely messed up to count since the organization of the *nse-4(tm7141)* germline is compromised.

NSE-4 localizes on the chromosome in all zones of the germline (Figure 7A), which showed a similar distribution to NSE-1, SMC-5, and SMC-6 in *C. elegans* (Bickel et al., 2010). The architecture of the SMC5/6 complex varies between organisms, and our current knowledge is based on studies in yeast [60,63,87,88,89], with substantial contributions from plants [61], humans [62], and *C. elegans* [25,45,54]. As SMC-5/SMC-6 together with NSE-4 kleisin serves as both structural and functional core of the SMC5/6 complex [7], we examined GFP::NSE-1 in *nse-4*, *smc-5* and *smc-6* mutants. These mutations completely delocalized NSE-1 from the complex, translocating it out of the nucleus into the cytoplasm (Figure 7C). Compared to the *nse-4(tm7158)*, the translocated NSE-1 seemed to aggregate more in *nse-4(tm7141)*, which could be related to the relatively smaller gonad. Collectively, our data demonstrates the importance of the *C. elegans nse-4* in the repair of DNA damage and the maintenance of genome integrity. This study provides additional insight into the role of NSE-4 and the architecture of the SMC-5/6 complex in *C. elegans*.

## 4. Materials and Methods

### 4.1. Worm Strains and Growth Condition

Worms were maintained at 20 °C on nematode growth medium (NGM) plates seeded with the food source OP50 (*Escherichia coli* strain) as previously described [90]. Worm strains were backcrossed to wild-type for at least five rounds before use. The *C. elegans* strains used in this study include: *N2* Bristol (wild-type), *smc-5(ok2421)*, *smc-6(ok3294)*, *nse-4(tm7141)*, *nse-4(tm7158)*, *clk-2(mn159)*, *brc-1(tm1145)*, *lig-4(rb873)*, *mus-81(tm1937)*, *polh-1(lf31)*, *syp-2(ok307)*, *nse-1::GFP (wsh1)*, *ced-1::GFP(smIs34)*, and *GFP::cosa-1(av630)*. The strains we generated as part of this study include: *smc-5(ok2421);syp-2(ok307)/nT1*, *smc-6(ok3294);syp-2(ok307)/nT1*, *nse-4(tm7141);syp-2(ok307)/nT1*, *nse-4(tm7158);syp-2(ok307)/nT1*, *ced-1::GFP(smIs34);smc-5(ok2421)/mln*, *ced-1::GFP(smIs34);smc-6/mln*, *ced-1::GFP(smIs34);tm7141/nT1*, *ced-1::GFP(smIs34);nse-4(tm7158/nT1)*, *nse-1::GFP(wsh1);nse-4(tm7141)/nT1*, *nse-1::GFP(wsh1);nse-4(tm7158)/nT1*, *GFP::cosa-1(av630);nse-4(tm7141)/nT1*, and *GFP::cosa-1(av630);nse-4(tm7158)/nT1*.

### 4.2. Phenotypic Assays (Brood-Size, Progeny Viability and Male Frequency)

For the brood-size assay of each strain, L4 hermaphrodites were picked into NGM plates (1 worm per plate) seeded in the center with OP50. The worms were transferred into freshly seeded NGM plates at every 12 h interval and the total eggs laid in the previous plate were counted. This was repeated for each worm until egg-laying ceased, and the total number of fertilized eggs laid throughout the fertile cycle for each strain was analyzed. Following 24 h after eggs are laid, the percentage of hatched eggs relative to the brood-size was used to quantify the progeny viability. After 72 h, when progeny have grown to a distinguishable stage, we counted the males and calculated the frequency as the percentage of males relative to number of animals that are alive.

### 4.3. Genotoxic Treatment and Animal Developmental Scoring

L1 developmental assays on worms treated with MMS, cisplatin, and HU were performed as described [31,32]. L1 worms were filtered out in M9 using 11 µm nylon net filters (Millipore) and treated with the indicated DNA damage agents in quadruplicates. For the MMS and cisplatin sensitivity assays, the worms were treated at the indicated doses for 16 h, while for HU, L1 worms were treated for 20 h. After the stipulated exposure time, the worms in each tube were washed with M9, dispensed onto an NGM agar plate seeded with OP50, and allowed to recover for 48 h at 20 °C. The developmental stages of the worms were scored under a stereomicroscope. Each experiment was repeated independently thrice.

### 4.4. Genotoxic Treatment and Progeny Viability Scoring

For the animal viability assays, at least 50 L1/L4 stage worms were treated with the reported doses of the indicated genotoxic agents (MMS, cisplatin, and HU) [31,32]. For the MMS and cisplatin sensitivity assays, the worms were treated at different doses for 16 h, while for HU, the worms were exposed for 20 h. Afterwards, the worms were allowed to recover for 72 h and 24 h for the L1 and L4 worms, respectively. Before viability scoring was completed as follows, we plated 5 adult worms from each strain and drug dose to lay eggs for 6–8 h on NGM plates freshly seeded with OP50. The worms were removed after the allowed egg-laying time, and the number of eggs laid in each plate was counted. The number of dead eggs was counted after 24 h. Progeny viability was then calculated as the percentage of hatched eggs to the total number of eggs laid. Each experiment was repeated independently thrice.

### 4.5. Worm Total RNA Extraction and RT-qPCR

Thirty (30) adult worms were used for total RNA extraction with TransZol Up Plus RNA kit according to manufacturer’s instructions. Then HiScript^®^ Ⅲ RT SurperMix for qPCR (Vazyme) was used for cDNA synthesis according to manufacturer instructions. The primer sequences of selected are shown as CCTCAACCTCTTCGGATCTT and TGCTCATCTCAGAGTCATCAA for *egl-1*, GCTCCCTGTTTATCACTTCTC and CTGGCATACGTCTTGAATCC for *ced-13*, AAGATCTATTGTTCTACCAGGC and CTTGAACTTCTTGTCCTTGAC for *tbg-1*. Each RT-qPCR was performed in a final volume of 20 µL, including 10 μL of 2 × ChamQ Universal SYBR qPCR Master Mix (Vazyme), 0.4 μL each of 10 μM forward and reverse primers, 2.0 μL of cDNA, and 7.2 μL of sterile water. Reaction procedures were as follows: initial denaturation for 30 s at 95 °C, followed by 40 cycles of 10 s at 95 °C and 30 s at 60 °C. Then, the melting curve program was set at 95 °C for 15 s, 60 °C for 1 min, 95 °C for 15 s. The fluorescence signal was measured at the end of each extension step at 80 °C. Relative expression levels of the tested genes were calculated using 2−△△CT method [91].

### 4.6. Construction of gfp::3xflag::nse-4 Strain and Microinjection

The CRISPR/Cas9 method was used to construct the *gfp::3xflag::nse-4* strain with green fluorescent labeling. Firstly, the *nse-4* sgRNA recognition site was selected near to the start codon of the *nse-4* gene, and the template of pU6::*nse-4* sgRNA, which drives *nse-4* sgRNA, was constructed by fusion PCR. At the same time, the *gfp::3xflag::nse-4* fusion template was constructed by fusion PCR as follows: The *gfp::tev-3xflag* DNA sequence was amplified from *rad54b::gfp::tev-3xflag* worm DNA. The *gfp::tev-3xflag* plus 27bp linker was inserted in-between the promoter and start codon of the *nse-4* gene. Then, the Cas9 plasmid, pU6::*nse-4* sgRNA DNA template, *gfp::3xflag::nse-4* fusion template, and screening markers were microinjected into the gonadal cells of nematodes, and the nematode progeny with screening markers were screened. Finally, PCR identification, sequencing analysis, and fluorescence signal detection were used to screen out the *gfp::3xflag::nse-4* strain. The strain was backcrossed to wild-type 4 times before use.

### 4.7. Cytological Preparations and Staining

Gonad extraction was performed according to the method previously described [32], with little optimization as described here. Paraformaldehyde-treated germline on cover slip was gently placed onto the poly-L-lysine slide, incubated for 5 min at room temperature (RT), and then in liquid nitrogen for a few minutes. The cover slip was then removed quickly, and the slide incubated in a jar of acetone/methanol (50%/50%) for 10 min. All DAPI staining was done using DAPI (100ng/mL) for 5 min and mounted using mounting medium and coverslip. The edges of the cover slip were sealed using nail polish. For RAD-51 antibody staining, after slides were incubated in a jar of acetone/methanol (50%/50%) for 10 min, they were washed three times (3×) for 10 min each in 0.3% Triton (Triton^®^ X-100) PBS buffer, and further washed once in PBT for 10 min. To pre-block the germline, 2–3 drops of Image enhancer (Invitrogen) were added and then incubated at RT for 15–20 min in a humid box. Next the Image enhancer was washed off by submerging the slide for a few seconds in PBST (PBS + 0.1% Tween 20). The slides were then washed three times in PBST for ten minutes each before being blocked for 20–30 min in a BSA (3%) + PBST solution. Next, we added 35–40 µL of 0.3% rabbit anti-RAD-51 antibody in 3% BSA to the sample, covered with a small piece of parafilm and incubated overnight at 4 °C. The antibody was washed off 4× in a jar of PBST for 10 min each, allowing the parafilm to fall off by itself. We then added 35–40 µL of 0.13% Goat Anti-Rabbit IgG (H+L) (A11034) in 3% BSA to the sample, covered with a parafilm and incubated for 2 h in a humid dark box at RT. The slides were washed 4× with PBST for 10 min each. Gonad samples were then counter-stained with DAPI as mentioned previously. The gonads were mounted using mounting medium and coverslip. The edges of the cover slip were sealed using nail polish.

### 4.8. Microscopy Procedures

The Motic^®^ SMZ-168 Stereo Zoom Dissecting Microscope was used for worm phenotypic analysis. Homozygous mutants were selected from heterozygous worms with balancers using Olympus SZX2-ILLB microscope. All imaging for statistical analysis was done using Leica DM6 B upright microscope with the Leica DFC7000 T camera and LAS X Software, with the exception of RAD-51 foci, which was done using Zeiss LSM800 confocal microscope with Airyscan. All whole germline single layer images were captured using a Zeiss LSM800 confocal microscope with 10× objective at a scale bar of 50 μm, except for *tm7141;nse-1::gfp* with a remarkably small germline in which 40× objective with oil immersion was used at a scale bar of 10 μm. All other images were captured using a Zeiss confocal microscope LSM 800 with Airyscan at 63× objective with oil immersion (scale bar = 10 μm). Oocytes were scored for statistical analysis using Leica DM6 B at 100× objective with oil immersion. RAD-51 foci scoring was carried out with Z-stack images using 488 filter of Zeiss confocal microscope LSM 800 with Airyscan at 63X objective with oil immersion (scale bar = 10 μm). The RAD-51 fluorescence intensity for each gonad was normalized to the gonad rachis background of each gonad set using the wild-type.

### 4.9. Statistical Analysis

Statistical differences in the data obtained were determined using one-way ANOVA with Fisher’s LSD, two-way ANOVA with Dunnett’s multiple comparisons, and two-tailed chi-square Fisher’s exact test for different experiments, as specified in the corresponding figure legends. The confidence level was indicated by the number of asterisks, where *p* > 0.05 (ns), * *p* < 0.05, ** *p* < 0.01, and **** *p* < 0.0001. For the unpaired two-tailed student’s *t*-test, the confidence level was set at *p* > 0.05 (ns), * *p* ≤ 0.05 and ** *p* ≤ 0.01. In all cases, bars with error bars represent means ± S. E. M.

## 5. Conclusions

Here, the mutants of *nse-4* exhibited serious phenotypic, chromosomal, developmental, and DNA repair defects. Some chromosomes appeared to be merged and distorted, possibly due to errors in repair and segregation. The sensitivity of the *nse-4* mutants to genotoxic stress, including MMS, HU, and cisplatin, points to its involvement in DNA repair. The increased RAD-51 foci in the mutants further confirms defects in meiotic repair, which, however, seemed not to affect other critical meiotic processes such as cross-over. The disrupted inter-sister recombination suggests that *nse-4* is essential for accurate progression of meiotic events. All these abnormalities in the *nse-4* mutants produced increased apoptosis that acted through the CED-13 and EGL-1 pathways. We further provide additional evidence for the interaction between the members of the smc-5/6 complex, where we showed that disrupting key members of SMC-5/6 complex, including *nse-4*, *smc-5*, and *smc-6*, delocalized the NSE-1 from the chromosomes, which translocated to the cytoplasm. Taken together, our study showed that NSE-4 is essential for genome stability and DNA repair in *C. elegans*.

## Data Availability

Not applicable.

## Supplementary Materials

The following supporting information can be downloaded at: https://www.mdpi.com/article/10.3390/ijms23137202/s1.
