# Supplementary material for: Loss of NSE-4 Perturbs Genome Stability and DNA Repair in Caenorhabditis elegans
Source: Int J Mol Sci. 2022 Jun 29;23(13):7202. doi: 10.3390/ijms23137202 (PMC9266361; doi:10.3390/ijms23137202)
Supplement: Supplementary file 1 [file ijms-23-07202-s001.zip › ijms-1719598-supplementary.pdf]

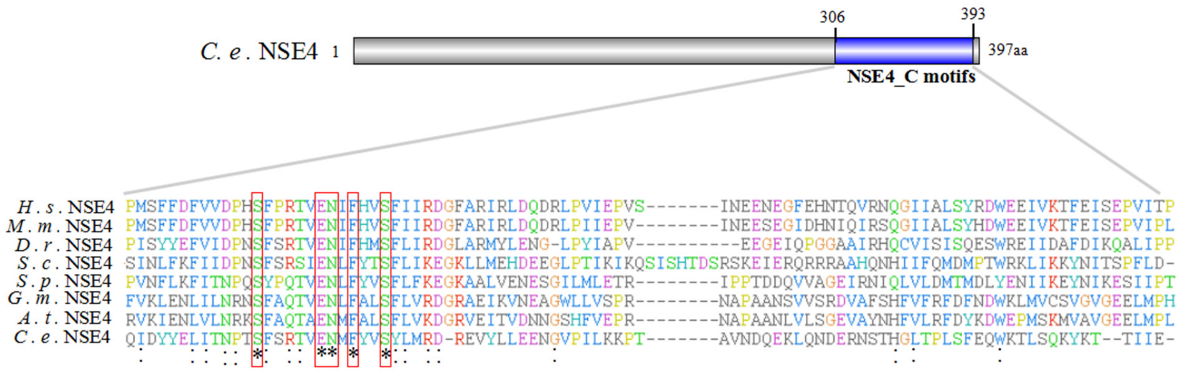

Figure S1. NSE-4 is conserved in *C. elegans*. Amino acid sequence alignment of the NSE4_C motifs in different species, including *Caenorhabditis elegans* NP_502167.2 (*C.e*), *Homo sapiens* XP_005269986.1 (*H.s*), *Danio rerio* NP_001018359.1 (*D.r*), *Mus musculus* NP_001156327.1 (*M.m*), *Saccharomyces cerevisiae* S288C NP_010178.1 (*S.c*), *Schizosaccharomyces pombe* NP_001018837.1 (*S.p*), *Glycine max* XP_003520535.1 (*G.m*), *Arabidopsis thaliana* NP_188712.4 (*A.t*) are presented. "*" represents identical amino acids; "**:**" represents similar amino acids; and "—" represents missing amino acids.

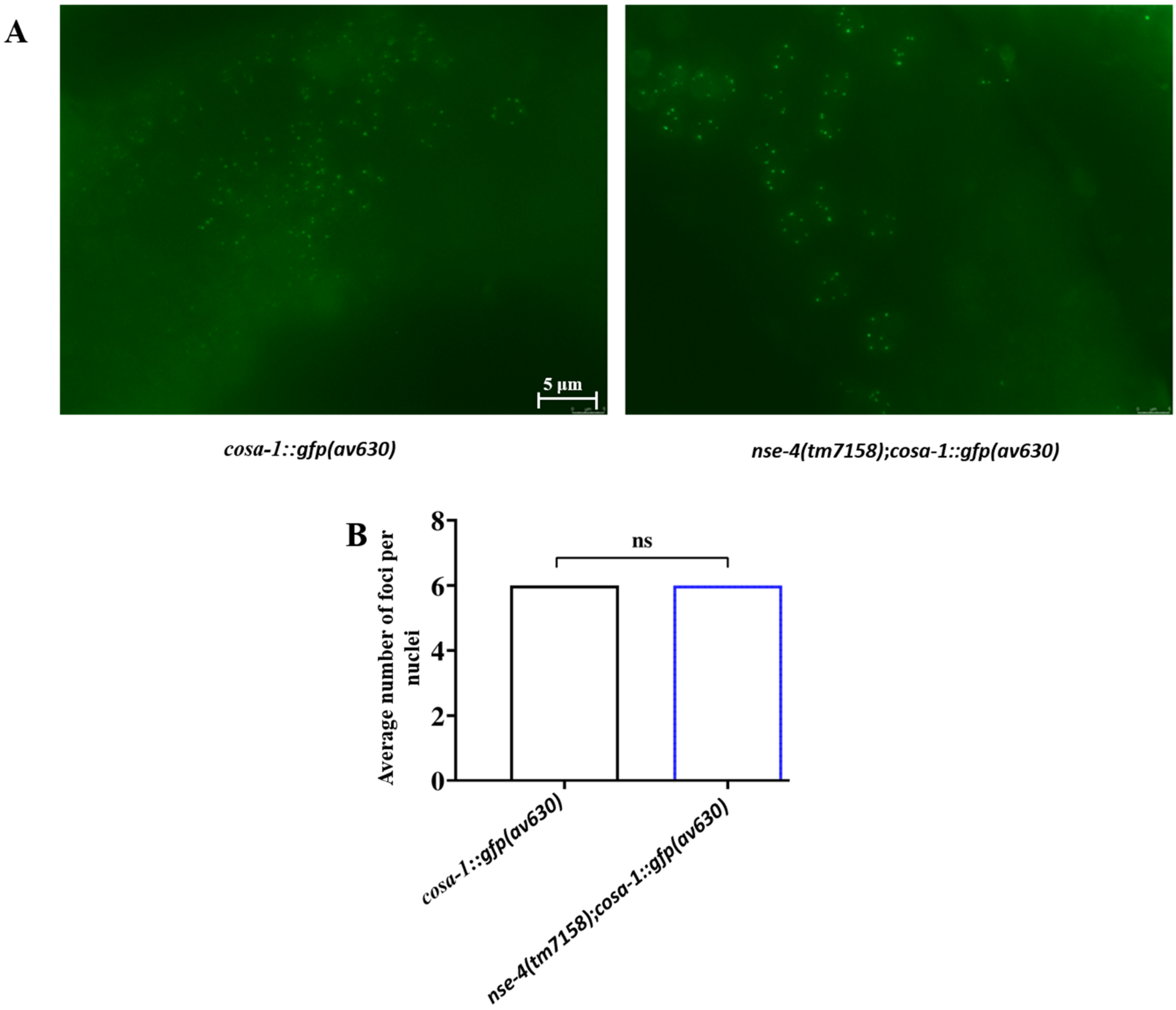

Figure S2. Mutations in *nse-4* did not result in any observable changes in the number and distribution of the cross-over factor COSA-1 as compared with wild-type. (A) Micrographs of the *gfp::cosa-1(av630)* and *nse-4(tm7158);cosa-1::gfp(av630)*. (**B**) Quantification of foci in *gfp::cosa-1(av630)* and *nse-4(tm7158);cosa-1::gfp(av630)*. Images were captured using Leica DM6 B at 100X objective with oil immersion (scale bar = 10 µm).

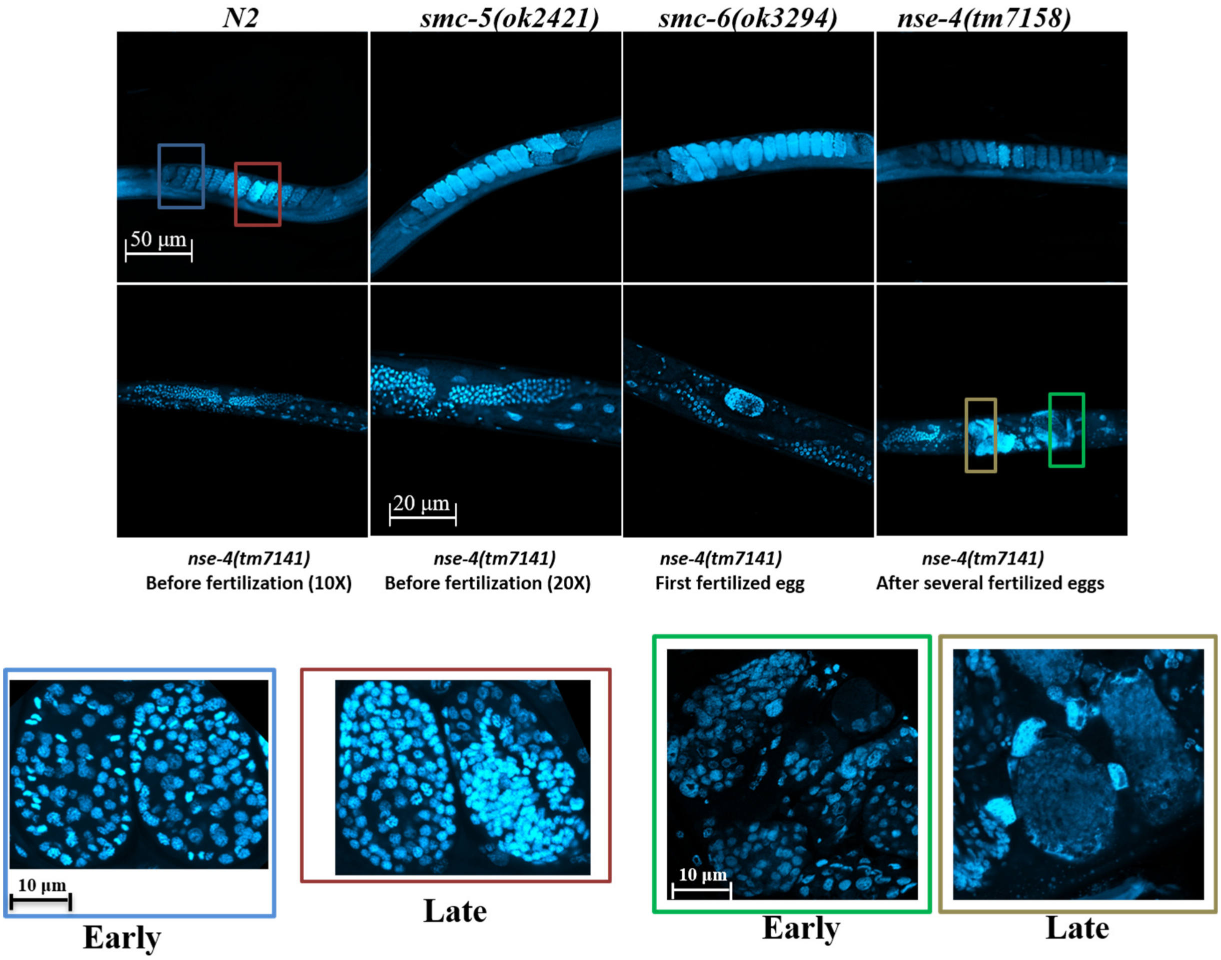

Figure S3. The gonad structure of gravid young adult worms showing the embryo arrangements. Egg-laying young adults were stained with DAPI to show the arrangement of the fertilized eggs. Wild-type, *smc-5(ok2421)*, *smc-6(ok3294)*, and *nse-4(tm7158)* mutants showed orderly arranged fertilized eggs. *Nse-4(tm7141)* on the contrary showed a disorganized arrangement after fertilization, showing a mitotic developmental defect. Images were captured using Zeiss LSM800 confocal microscope using 10X objective at a scale bar of 50 µm, except for *nse-4(tm7141)* which was captured using 20X objective at a scale bar of 20 µm due to its comparatively smaller germline. Blue, red, green, and yellow boxes represent the early and late stages of embryo development in the intact worm. Their corresponding enlargement is shown in the larger boxes.

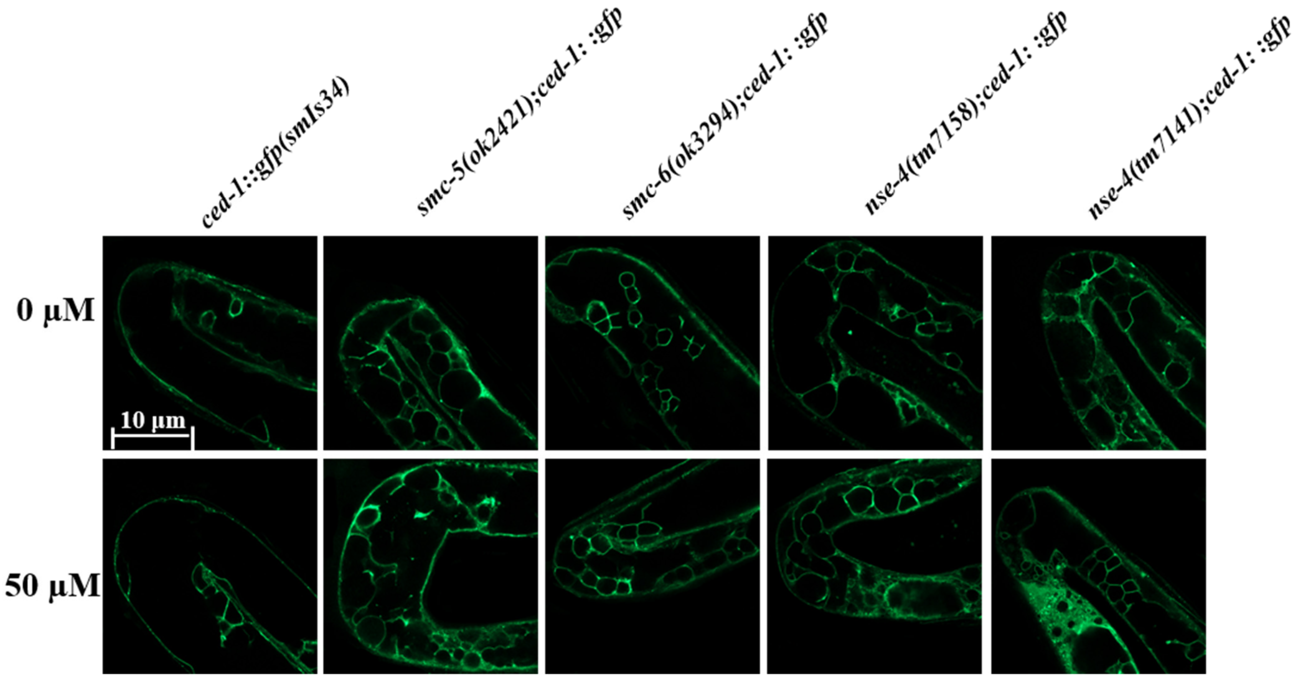

Figure S4. Cisplatin treatment caused increased rings of GFP fluorescence (CED-1::GFP) surround apoptotic cells in the gonad arm of worms. All images were captured using Zeiss confocal microscope LSM 800 with Airyscan at 63X objective with oil immersion (scale bar = 10 μm).

Table S1. Phenotypic analysis of the strains

Total number (n)	N2	*smc-5(ok2421)*	*smc-6(ok3294)*	*nse-4(tm7141)*	*nse-4(tm7158)*
Brood-size (worms)	22	22	20	18	21
Progeny viability (eggs)	5810	3120	1844	175	2502
Male frequency (progeny)	5733	2717	1066	163	1861

Table S2. Total number (n) of worms scored for developmental stages at 48 hours post MMS treatment at L1

Strains	Doses		
	**0 mM**	**0.15 mM**	**0.4 mM**
wild-type *(N2)*	2111	2039	2116
clk-2	1975	2212	2161
mus-81	1919	1965	1688
smc-5(ok2421)	2602	2688	2421
smc-6(0k3294)	1890	2013	2050
nse-4(tm7158)	1416	1493	1417

Table S3. Total number of worms scored for developmental stages at 48 hours post HU treatment at L1

Strains	Doses			
	**0 mM**	**5 mM**	**10 mM**	**15 mM**
wild-type *(N2)*	2525	2538	2634	2350
clk-2	2061	2324	2422	2568
polh-1	3003	2977	3202	3110
smc-5(ok2421)	2151	1998	1959	2014
smc-6(0k3294)	1790	1902	2067	2039
nse-4(tm7158)	1734	1720	1689	1925

Table S4. Total number of worms scored for developmental stages at 48 hours post cisplatin treatment at L1

Strains	Doses			
	**0 μM**	**20 μM**	**60 μM**	**100 μM**
wild-type *(N2)*	665	822	786	863
lig-4 (rb873)	1033	972	965	875
brc-1(tm1145)	675	625	760	652
smc-5(ok2421)	953	963	1134	1087
smc-6(0k3294)	666	561	698	644
nse-4(tm7158)	831	982	999	992

Table S5. Total eggs laid and viability (%) of worms at 72 hours post MMS treatment at L1

Strains	Doses		
	**0 mM**	**0.15 mM**	**0.4 mM**
wild-type*(N2)*	1686 (98.3%) $^{a, 1}$	1912 (98.5%) $^{a, 1}$	1877 (98.5%) $^{a, 1}$
clk-2	1222 (92.4%) $^{a, 1}$	939 (77.6%) $^{b, 2}$	324 (10.9%) $^{b, 3}$
mus-81	1724 (88.5%) $^{a, b, 1}$	463 (29.7%) $^{c, 2}$	18 (0%) $^{b, 3}$
smc-5(ok2421)	1162 (78.1%) $^{b, c, 1}$	29 (5.1%) $^{d, 2}$	9 (1.9%) $^{b, 2}$
smc-6(0k3294)	1223 (74.8%) $^{c, 1}$	52 (0%) $^{d, 2}$	0 (0%) $^{b, 2}$
nse-4(tm7158)	987 (77.5%) $^{c, 1}$	0 (0%) $^{d, 2}$	0 (0%) $^{b, 2}$

- Letter superscripts denote statistical difference in worm viability (%) between strains (within a column). Same letter superscripts indicate nonsignificant (ns) difference, and different letter superscripts denote significant difference.
- Numeric superscripts denote statistical difference in worm viability (%) between doses (across a row). Same numeric superscripts indicate nonsignificant (ns) difference, and different letter superscripts denote significant difference.

Table S6. Total eggs laid and viability (%) of worms at 72 hours post HU treatment at L1

Strains	Doses		
	**0 mM**	**5 mM**	**10 mM**
wild-type*(N2)*	2075 (98.8%) $^{a, 1}$	1906 (97.5%) $^{a, 1}$	1508 (98.2%) $^{a, 1}$
clk-2	1344 (95.5%) $^{a, b, 1}$	858 (93.5%) $^{a, 1}$	281 (88.9%) $^{a, 1}$
polh-1	1890 (97.7%) $^{a, b, 1}$	2113 (97.0%) $^{a, 1}$	1343 (95.1%) $^{a, 1}$
smc-5(ok2421)	1344 (73.2%) $^{b, c, 1}$	1081 (64.1%) $^{b, 1}$	375 (47.7%) $^{b, 2}$
smc-6(0k3294)	1719 (71.1%) $^{c, 1}$	1227 (65.5%) $^{b, 1}$	521 (61.5%) $^{b, 1}$
nse-4(tm7158)	1249 (71.0%) $^{c, 1}$	1095 (62.4%) $^{b, 1}$	401 (41.5%) $^{b, 2}$

- Letter superscripts denote statistical difference in worm viability (%) between strains (within a column). Same letter superscripts indicate nonsignificant (ns) difference, and different letter superscripts denote significant difference.
- Numeric superscripts denote statistical difference in worm viability (%) between doses (across a row). Same numeric superscripts indicate nonsignificant (ns) difference, and different letter superscripts denote significant difference.

Table S7. Total eggs laid and viability (%) of worms at 24 hours post MMS treatment at L4

**Strains**	**Doses**		
	**0 mM**	**0.15 mM**	**0.4 mM**
wild-type*(N2)*	1069 (97.4%) [a, 1]	1041 (95.5%) [a, 1]	1037 (94.2%) [a, 1]
clk-2	661 (77.1%) [c, d,1]	652 (75.5%) [b, 1]	392 (64.7%) [b, 2]
mus-81	1036 (89.1%) [a, b, 1]	1079 (1.6%) [d, 2]	593 (0%) [d, 2]
smc-5(ok2421)	1083 (85.7%) [b, c, 1]	1074 (80.1%) [b, 1, 2]	770 (72.2%) [b, 2]
smc-6(0k3294)	879 (58.1%) [e, 1]	853 (55.8%) [c, 1]	502 (38.7%) [c, 2]
nse-4(tm7158)	895 (71.4%) [d, 1]	759 (62.4%) [c, 2]	558 (33.2%) [c, 3]

- Letter superscripts denote statistical difference in worm viability (%) between strains (within a column). Same letter superscripts indicate nonsignificant (ns) difference, and different letter superscripts denote significant difference.
- Numeric superscripts denote statistical difference in worm viability (%) between doses (across a row). Same numeric superscripts indicate nonsignificant (ns) difference, and different letter superscripts denote significant difference.

Table S8. Total eggs laid and viability (%) of worms at 24 hours post cisplatin treatment at L4

	**0 μM**	**50 μM**	**100 μM**	**200 μM**	**400 μM**
wild-type*(N2)*	2251 (97.9%) [a, 1]	1946 (85.1%) [a, 1]	1662 (63.8%) [a, 2]	1068 (42.8%) [a, 3]	613 (18.2%) [a, 4]
xpf-1	2085 (79.8%) [b, 1]	1545 (0.2%) [c, 2]	1111 (0%) [c, 2]	486 (0%) [c, 2]	112 (0%) [b, 2]
brc-1(tm1145)	1942 (96.0%) [a, 1]	1595 (85.0%) [a, 1]	1573 (67.6%) [a, 2]	865 (18.0%) [b, 3]	257 (0.4%) [b, 4]
smc-5(ok2421)	1789 (84.7%) [a, b, 1]	1264 (46.8%) [b, 2]	924 (28.5%) [b, 3]	639 (16.8%) [b, 3, 4]	465 (3.9%) [a, b, 4]
smc-6(0k3294)	1431 (73.7%) [b, 1]	725 (1.2%) [c, 2]	373 (0.2%) [c, 2]	273 (0%) [c, 2]	150 (0%) [b, 2]
nse-4(tm7158)	1606 (79.3%) [b, 1]	665 (1.1%) [c, 2]	405 (0%) [c, 2]	301 (0%) [c, 2]	95 (0%) [b, 2]

- Letter superscripts denote statistical difference in worm viability (%) between strains (within a column). Same letter superscripts indicate nonsignificant (ns) difference, and different letter superscripts denote significant difference.
- Numeric superscripts denote statistical difference in worm viability (%) between doses (across a row). Same numeric superscripts indicate nonsignificant (ns) difference, and different letter superscripts denote significant difference.

Table S9. Total number (n) of nuclei scored for RAD-51 foci for all the genotypes investigated

	**Mitotic zone**	**Transition zone**	**Early-pachytene**	**Mid-pachytene**	**Late-pachytene**	**Diplotene**	**Diakinesis**
wild-type*(N2)*	469	210	319	299	225	50	50
smc-5(ok2421)	269	198	274	248	320	50	50
smc-6(ok3294)	230	379	295	249	288	50	50
nse-4(tm7158)	336	206	317	282	236	50	50
nse-4(tm7141)	227	102		283*		50	50

*Due to the absence of a clearly differentiated early, mid- and late pachytene in the nse-4(tm7141), the RAD-51 foci in the entire pachytene were quantified together.

Table S10. Significance of *egl-1* relative expression between different stains

Strains	(n)	Significance
N2 (0µm) vs *smc-5(ok2421)* (0µm)	6 vs 5	** p < 0.01
N2 (0µm) vs *smc-6(0k3294)* (0µm)	6 vs5	** p < 0.01
N2 (0µm) vs *nse-4(tm7158)* (0µm)	6 vs 4	** p < 0.01
N2 (0µm) vs *nse-4(tm7141)* (0µm)	6 vs 3	** p < 0.01
N2 (0µm) vs *xpf-1(tm2842)* (0µm)	6 vs5	** p < 0.01
N2 (0µm) vs *brc-1(tm1145)* (0µm)	6 vs5	ns p > 0.05
N2 (0µm) vs *lig-4(rb873)* (0µm)	6 vs 6	ns p > 0.05
N2 (0µm) vs N2 (50µm)	6 vs 5	** p < 0.01
smc-5(ok2421) (0µm) vs *smc-5(ok2421)* (50µm)	5 vs 5	ns p > 0.05
smc-6(0k3294) (0µm) vs *smc-6(0k3294)* (50µm)	5 vs 5	* p < 0.05
nse-4(tm7158) (0µm) vs *nse-4(tm7158)* (50µm)	4 vs 4	ns p > 0.05
nse-4(tm7141) (0µm) vs *nse-4(tm7141)* (50µm)	3 vs 3	ns p > 0.05
xpf-1(tm2842) (0µm) vs *xpf-1(tm2842)* (50µm)	5 vs 4	ns p > 0.05
brc-1(tm1145) (0µm) vs *brc-1(tm1145)* (50µm)	5 vs6	** p<0.01
lig-4(rb873) (0µm) vs *lig-4(rb873)* (50µm)	6 vs 5	**p<0.01

Table S11. Significance of *ced-13* relative expression between different stains

Stains	(n)	significance
N2 (0µm) vs *smc-5(ok2421)* (0µm)	6 vs 4	** p < 0.01
N2 (0µm) vs *smc-6(0k3294)* (0µm)	6 vs 4	** p < 0.01
N2 (0µm) vs *nse-4(tm7158)* (0µm)	6 vs 5	** p < 0.01
N2 (0µm) vs *nse-4(tm7141)* (0µm)	6 vs 3	** p < 0.01
N2 (0µm) vs *xpf-1(tm2842)* (0µm)	6 vs 6	** p < 0.01
N2 (0µm) vs *brc-1(tm1145)* (0µm)	6 vs 6	**p < 0.01
N2 (0µm) vs *lig-4(rb873)* (0µm)	6 vs 6	ns p > 0.05
N2 (0µm) vs N2 (50µm)	6 vs 6	** p < 0.01
smc-5(ok2421) (0µm) vs *smc-5(ok2421)* (50µm)	4 vs 4	ns P > 0.05
smc-6(0k3294) (0µm) vs *smc-6(0k3294)* (50µm)	4 vs 4	ns P > 0.05
nse-4(tm7158) (0µm) vs *nse-4(tm7158)* (50µm)	5 vs 4	ns P > 0.05
nse-4(tm7141) (0µm) vs *nse-4(tm7141)* (50µm)	3 vs 3	ns P > 0.05
xpf-1(tm2842) (0µm) vs *xpf-1(tm2842)* (50µm)	6 vs 6	ns P > 0.05
brc-1(tm1145) (0µm) vs *brc-1(tm1145)* (50µm)	6 vs6	** p < 0.01
lig-4(rb873) (0µm) vs *lig-4(rb873)* (50µm)	5 vs 5	** p< 0.01
